# Temporal requirement of dystroglycan glycosylation during brain development and rescue of severe cortical dysplasia via gene delivery in the fetal stage

**DOI:** 10.1093/hmg/ddy032

**Published:** 2018-01-19

**Authors:** Atsushi Sudo, Motoi Kanagawa, Mai Kondo, Chiyomi Ito, Kazuhiro Kobayashi, Mitsuharu Endo, Yasuhiro Minami, Atsu Aiba, Tatsushi Toda

**Affiliations:** 1 Division of Neurology/Molecular Brain Science, Kobe University Graduate School of Medicine, Kobe 650-0017, Japan; 2 Division of Cell Physiology, Kobe University Graduate School of Medicine, Kobe 650-0017, Japan; 3 Laboratory of Animal Resources, Center for Disease Biology and Integrative Medicine, The University of Tokyo, Tokyo 113-0033, Japan; 4 Department of Neurology, Graduate School of Medicine, The University of Tokyo, Tokyo 113-0033, Japan

## Abstract

Congenital muscular dystrophies (CMDs) are characterized by progressive weakness and degeneration of skeletal muscle. In several forms of CMD, abnormal glycosylation of α-dystroglycan (α-DG) results in conditions collectively known as dystroglycanopathies, which are associated with central nervous system involvement. We recently demonstrated that *fukutin*, the gene responsible for Fukuyama congenital muscular dystrophy, encodes the ribitol-phosphate transferase essential for dystroglycan function. Brain pathology in patients with dystroglycanopathy typically includes cobblestone lissencephaly, mental retardation, and refractory epilepsy; however, some patients exhibit average intelligence, with few or almost no structural defects. Currently, there is no effective treatment for dystroglycanopathy, and the mechanisms underlying the generation of this broad clinical spectrum remain unknown. Here, we analysed four distinct mouse models of dystroglycanopathy: two brain-selective *fukutin* conditional knockout strains (neuronal stem cell-selective *Nestin*-*fukutin*-cKO and forebrain-selective *Emx1*-*fukutin*-cKO), a *Fukutin*^Hp^ strain with the founder retrotransposal insertion in the *fukutin* gene, and a spontaneous *Large*-mutant *Large*^myd^ strain. These models exhibit variations in the severity of brain pathology, replicating the clinical heterogeneity of dystroglycanopathy. Immunofluorescence analysis of the developing cortex suggested that residual glycosylation of α-DG at embryonic day 13.5 (E13.5), when cortical dysplasia is not yet apparent, may contribute to subsequent phenotypic heterogeneity. Surprisingly, delivery of *fukutin* or *Large* into the brains of mice at E12.5 prevented severe brain malformation in *Emx1*-*fukutin*-cKO and *Large*^myd/myd^ mice, respectively. These findings indicate that spatiotemporal persistence of functionally glycosylated α-DG may be crucial for brain development and modulation of glycosylation during the fetal stage could be a potential therapeutic strategy for dystroglycanopathy.

## Introduction

Post-translational modification refers to the chemical modification of proteins following translation—a process that regulates many functions of the proteins involved. Glycosylation is among the most critical post-translational modifications, and abnormal glycosylation is often associated with human diseases such as congenital muscular dystrophy (CMD). While CMD refers to a clinically and genetically heterogeneous group of inherited muscle disorders, a subset of CMDs, including Walker-Warburg syndrome (WWS), muscle-eye-brain disease (MEB), and Fukuyama congenital muscular dystrophy (FCMD), are accompanied by cobblestone (type II) lissencephaly (1). Cobblestone lissencephaly is characterized by bumpy cortical surfaces and shallow sulci, and previous studies have shown that CMDs accompanied by cobblestone lissencephaly are caused by defective glycosylation of α-dystroglycan (α-DG). Therefore, these types of CMDs are now categorized as dystroglycanopathies (2,3), for which there is currently no effective treatment.

Dystroglycan (DG) is a component of the dystrophin-glycoprotein complex, which links the extracellular matrix to the intracellular actin cytoskeleton (4). DG is composed of α and β subunits, which are encoded by a single mRNA and cleaved into the two subunits during post-translational modification. The α-DG subunit binds to several extracellular matrix and synaptic proteins such as laminin, agrin, neurexin, and pikachurin (5–8). Proper *O*-mannosyl glycosylation of α-DG is required for these ligand-binding functions (9), and the loss of such functions due to abnormally glycosylated α-DG consequently affects various physiological processes associated with α-DG-ligand connections. DG provides structural support to the sarcolemma by connecting the basement membrane with the cytoskeleton in skeletal muscles, and disruption of this linkage is thought to cause various types of muscular dystrophy (10). In the developing cortex, abnormally glycosylated α-DG on radial glia is unable to maintain the integrity of the glia limitans-basement membrane complex (11–13). Protrusion of neurons into the subarachnoid space through breaches in this complex has been regarded as a major pathogenic process leading to polymicrogyria in cobblestone lissencephaly (14).

The primary cause of various forms of dystroglycanopathy involves the mutation of genes associated with *O*-glycosylation of α-DG. To date, at least 18 genes, including *fukutin*, fukutin-related protein (*FKRP*), isoprenoid synthase domain-containing protein (*ISPD*), and like-acetylglucosaminyltransferase (*LARGE*), have been identified as causative genes for dystroglycanopathy (15). *Fukutin* is the first dystroglycanopathy gene identified for FCMD, which is among the most common autosomal recessive disorders in Japan. FCMD is characterized by severe CMD associated with brain malformation and ocular abnormalities (16). Japanese patients with FCMD carry the founder 3’-UTR 3kb retrotransposal insertion in the *fukutin* gene (17). In such patients, mutations of this gene are either homozygous for the insertion or compound heterozygous with another deleterious mutation. Recently, we reported that the tandem ribitol 5-phosphate (Rbo5P) structure in the sugar chain of α-DG is required for synthesis of the ligand-binding moiety and that fukutin, FKRP, and ISPD are critical for this modification (18). Fukutin and FKRP are both Rbo5P transferases (18), while ISPD catalyzes CDP-ribitol synthesis as a CDP-ribitol pyrophosphorylase (18–20). The ligand-binding moiety, which is composed of repeating units of xylose and glucuronic acid, is synthesized by LARGE, a bifunctional glycosyltransferase with both xylosyltransferase and glucuronyltransferase activities (21). Mutation of the *LARGE* gene causes CMD type 1D (MDC1D), which is accompanied by severe mental retardation and brain malformation (22).

Although the biosynthetic pathway for functional α-DG has been elucidated, the mechanisms underlying the generation of the broad range of clinical dystroglycanopathy phenotypes remain unknown. Clinical variation ranges from very severe malformations accompanied by mental retardation to few or almost no structural defects accompanied by average intelligence. Research has indicated that mutations of the *fukutin* gene can cause three forms of the disease: dystroglycanopathy type A4 (the most severe form), type B4 (a less severe form without mental retardation), and type C4 (a milder limb-girdle form) (23). Although previous studies have suggested that clinical heterogeneity in patients with FCMD can be explained in part by differences in the types of mutations (24), some of which may influence the enzymatic activity of fukutin, the precise mechanism remains unclear.

The molecular and cellular pathomechanisms of clinical phenotypes in the skeletal muscle of patients with dystroglycanopathy have been relatively well-characterized, whereas comparatively less is known regarding brain pathology. Since brain abnormalities represent an important clinical feature of dystroglycanopathy, understanding the pathophysiological roles of α-DG glycosylation in the brain is necessary for elucidating factors underlying the pathogenesis and clinical heterogeneity of these disorders, and ultimately for developing an effective therapeutic strategy. In the present study, we analysed four distinct mouse models of dystroglycanopathy that replicate the heterogeneity of brain pathology in CMDs. Our results indicate that the state of glycosylation during brain development may influence the severity of subsequent brain pathology, suggesting that spatiotemporal persistence of functionally glycosylated α-DG is crucial for normal brain development during the fetal stage. Our findings also provide insight regarding the potential for therapeutic intervention during the fetal stage, which may prevent brain malformation in patients with dystroglycanopathy.

## Results

### Generation and characterization of brain-selective *Nestin*-*fukutin*-cKO mice

We initially aimed to elucidate the pathogenesis of brain abnormalities in dystroglycanopathies using mice with brain-selective conditional knockout of *fukutin* (*fukutin*-cKO mice) as a model. We previously reported the pathological analysis of *fukutin*-deficient chimeric mice (25,26), although chimeric rates were not identical among these mice, thus preventing precise interpretation of obtained results. To achieve our aim, we instead generated brain-selective *Nestin*-*fukutin*-cKO mice. We used a Cre-LoxP conditional knockout system (27) because homozygous germline disruption of the *fukutin* gene results in early embryonic lethality in mice, limiting studies during development (28).

**Figure 1.**
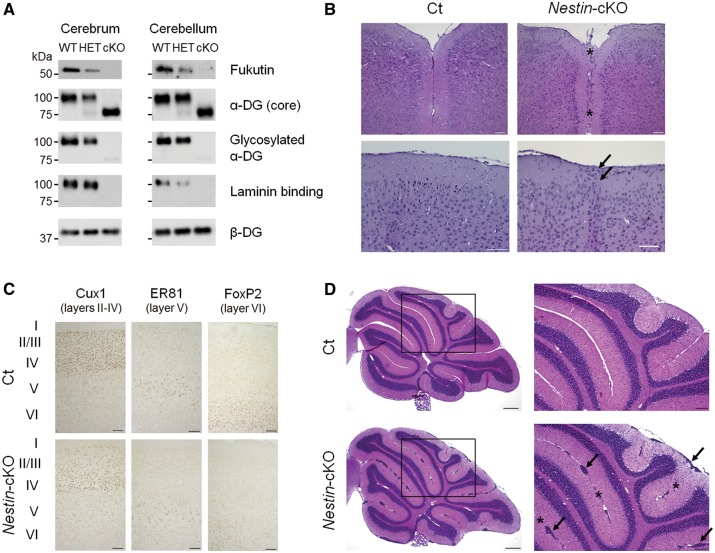
Generation and characterization of brain-selective *Nestin*-*fukutin*-cKO mice. (**A**) Western blotting analysis of endogenous fukutin expression and α-DG glycosylation in the adult cerebrum and cerebellum. Laminin-binding activity of α-DG was examined by a laminin overlay assay. We used β-DG as a loading control. (**B**) Mild focal abnormalities were observed in the cerebrum of adult *Nestin*-*fukutin*-cKO mice. Hematoxylin and eosin (H&E) staining revealed fusion of the cerebral hemispheres in cKO mice (asterisk). Ectopic cellular infiltration into layer I was observed in cKO mice (arrow). (**C**) Laminar organization of cerebral cortex. No obvious difference was detected between cKO mice and littermate controls. Cux1 was used as a marker of neurons in layers II-IV of cerebral cortex. ER81 and FoxP2 were used as markers of neurons in layer V or VI, respectively. (**D**) H&E staining of the cerebellum. Ectopic cells between adjacent cerebellar lobules and at the surface of cerebellar lobules were diffusely distributed in cKO mice (arrow). Cerebellar lobules were fused at many sites (asterisk). Scale bars = (B, C) 100 μm; (D) 300 μm (left column), 100 μm (right column). α-DG, alpha dystroglycan; Ct, control; cKO, conditional knockout.

First, we confirmed the loss of fukutin protein in the cerebrum and cerebellum of adult mice. Fukutin expression was the most abundant in wild-type (WT) mice, while expression was reduced and absent in heterozygous (HET) and cKO mice, respectively (Fig. 1A). Abnormal glycosylation of α-DG was indicated by decreased molecular weight, loss of immunoreactivity against α-DG (IIH6) antibody—which recognizes functionally glycosylated α-DG—and decreased laminin-binding activity (Fig. 1A). Histological examination revealed focal cortical dysplasia. Fused cerebral hemispheres and heterotopic cells in layer I of the cortex were observed in some cKO mice (Fig. 1B), while we also observed no apparent brain abnormalities in a few of cKO mice. With the exception of these lesions, the laminar organization of the cerebral cortex was essentially preserved (Fig. 1C). Previous studies have reported hippocampal dysplasia in other mouse models of dystroglycanopathies (12,29). However, in the present study, no apparent pathological changes were detected in the hippocampus (Supplementary Material, Fig. S1). Ectopic cells were diffusely present at many fusion sites between adjacent cerebellar lobules and at the surface of cerebellar lobules in cKO mice (Fig. 1D).

### Disruption of the glia limitans-basement membrane complex underlies cerebral and cerebellar malformation in *Nestin*-*fukutin*-cKO mice

**Figure 2.**
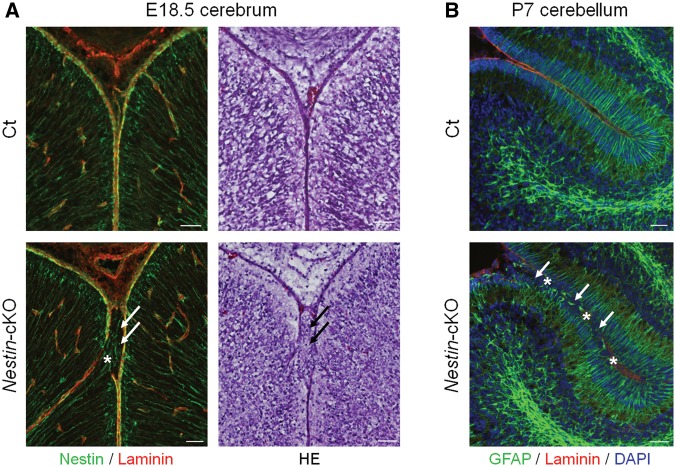
Damage of the basement membrane and disorganization of glial cells underlie cerebral and cerebellar malformations in *Nestin*-*fukutin*-cKO mice. (**A**) (Left column) Immunofluorescence analysis of the developing cortex at E18.5. Radial glia fibers were disorganized and extended into subarachnoid spaces (white arrow) through breaches in the basement membrane (asterisk) in cKO mice. In contrast, they had merged properly in littermate controls. Nestin and laminin were used as markers of radial glia or the basement membrane, respectively. (Right column) H&E staining of serial sections. Ectopic cells were observed at cerebral fissures (black arrow) in cKO mice. (**B**) Immunofluorescence analysis of the cerebellum at P7. Between adjacent cerebellar lobules, the basement membrane was not detected (asterisk), and many ectopic cells were observed (arrow) in cKO mice. Bergmann glia fibers were disorganized, and reactive gliosis was detected in such regions. In contrast, they had merged properly in littermate controls. GFAP was used as a marker of Bergmann glia. Scale bars = (A, B) 50 μm.

**Figure 3.**
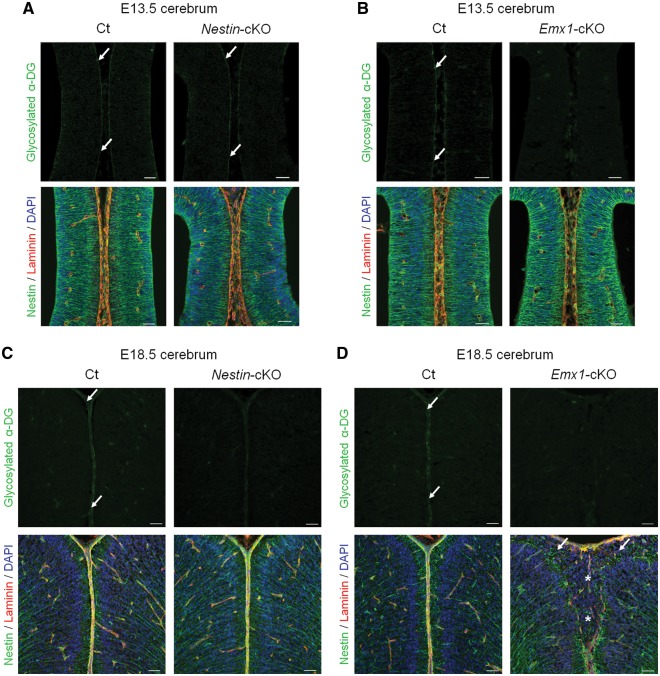
Dystroglycan glycosylation and phenotypic correlation during brain development in *fukutin*-cKO mice. (**A**, **B**) Immunofluorescence analysis of the developing cortex at E13.5. (A, upper panel) *Nestin*-*fukutin*-cKO mice demonstrated residual glycosylation of α-DG at the glia limitans as in littermate controls (arrow). (B, upper panel) Defective glycosylation of α-DG was observed in *Emx1*-*fukutin*-cKO mice. (A and B, lower panel) Both strains exhibited almost normal brain structure regardless of α-DG glycosylation state. (**C**, **D**) Immunofluorescence analysis of the developing cortex at E18.5. (C and D, upper panel) Both strains demonstrated defective glycosylation of α-DG in contrast to littermate controls. (C, lower panel) With the exception of focal cortical dysplasia within a limited region, the laminar organization of the cerebral cortex and basement membrane were preserved in *Nestin*-*fukutin*-cKO mice. (D, lower panel) Diffuse and severe brain malformations were observed in *Emx1*-*fukutin*-cKO mice, in contrast to very mild brain pathology in *Nestin*-*fukutin*-cKO mice. Cerebral hemispheres were widely fused (asterisk), and the glia limitans-basement membrane complex was diffusely dissociated in *Emx1*-*fukutin*-cKO mice. Many ectopic cells were observed in the subarachnoid space (arrow). Scale bars = (A, B, C, D) 50 μm.

Since neuronal migration in the cerebral cortex occurs during the embryonic stage, we next analysed embryonic brains in order to elucidate the pathogenesis of brain abnormalities. In the cerebral cortex, radial glia serve as neuronal and glial progenitors and provide scaffolding for neuronal radial migration during development (30). The processes of radial glia extend from the ventricular zone, forming foot processes that contact the basement membrane at the pial surface. In littermate controls, the basement membrane at the glia limitans was intact and had merged with radially oriented glial fibers (Fig. 2A). In a few of *Nestin*-*fukutin*-cKO mice, the basement membrane was discontinuous at the glia limitans. In such mice, radially oriented glial fibers at the glia limitans were disorganized and extended into the subarachnoid space, in which ectopic cells were observed (Fig. 2A and Supplementary Material, Fig. S2). With the exception of such lesions, the glia limitans-basement membrane complex was well preserved as shown in Figure 3C.

In contrast to that of the cerebral cortex, development of the cerebellum begins during the embryonic stage and continues after birth. Since previous reports have indicated that cerebellar pathology does not become evident until birth in mouse models of dystroglycanopathy (31), we examined the cerebellum at P7, an age at which active proliferation, differentiation, and migration of granule cells can be observed. In littermate controls, the basement membrane was intact and had merged with Bergmann glia fibers, which provide scaffolding for the radial migration of granule cells (32) (Fig. 2B). In contrast, the basement membrane was discontinuous at the glia limitans in *Nestin*-*fukutin*-cKO mice, and Bergmann glia fibers were retracted and disorganized in these regions (Fig. 2B). The pial basement membrane was not detected in regions where two cerebellar folia had completely fused.

### The glycosylation state of α-DG at E13.5 is associated with subsequent pathological severity in mouse models of dystroglycanopathy

Patients with FCMD demonstrate a range of phenotypes, such as a fusion of cerebral hemispheres, ectopic cells in the cerebral and cerebellar cortex, and overmigration of neurons through the breaches in the glia limitans-basement membrane complex (14,16). Although *Nestin*-*fukutin*-cKO mice developed using a Cre-LoxP conditional knockout system partially replicated these phenotypes, brain malformations in such mice were much milder than those in human patients with FCMD. Furthermore, a severe disorganization of cortical layers, occasionally observed in the brain of patients with FCMD, was not detected in *Nestin*-*fukutin*-cKO mice. Recent studies have indicated that *Nestin*-*Cre* transgenic mice may exhibit insufficient recombination with respect to early neural progenitors (33); therefore, we hypothesized that residual levels of normally glycosylated α-DG during the early stages of brain development may have prevented severe malformation. To test this hypothesis, we analysed another type of CNS-selective *fukutin*-cKO mouse (*Emx1*-*fukutin*-cKO) developed using *Emx1*-*Cre* knock-in mice, which has been reported to drive sufficient recombination of the target gene in dorsal telencephalic neural stem cells and achieve almost complete recombination as early as E12.5 (33,34). Since cortical dysplasia first becomes apparent at E14 in *fukutin*-deficient chimeric mice (26), we performed immunofluorescence analysis of the developing cortex in these two *fukutin*-cKO strains at E13.5 (equivalent to the preclinical stage) and E18.5, respectively.

**Figure 4.**
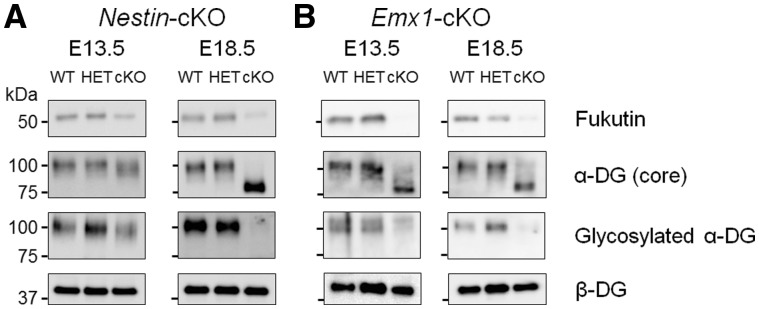
Western blotting analysis in the fetal brains of *fukutin*-cKO mice. (**A**) *Nestin*-*fukutin*-cKO and (**B**) *Emx1*-*fukutin*-cKO strains were analysed at E13.5 and E18.5, respectively. We used β-DG as a loading control.

Interestingly, *Nestin*-*fukutin*-cKO mice demonstrated residual levels of normally glycosylated α-DG on radial glia endfeet at E13.5 in spite of Cre expression driven by *Nestin* promoter beginning at E10.5 (Fig. 3A, upper panel). In contrast, normally glycosylated α-DG was not detected in the developing cortex of *Emx1*-*fukutin*-cKO mice at E13.5 (Fig. 3B, upper panel). Examination via light microscopy revealed no obvious structural defects in either mouse strain at this stage, regardless of α-DG glycosylation state (Fig. 3A and B, lower panel). In *Nestin*-*fukutin*-cKO mice, focal cortical dysplasia, such as ectopic cells at cerebral fissures (Fig. 2A) and the subarachnoid space (Supplementary Material, Fig. S2) was observed only within a limited region in a few of cKO mice. Essentially, the laminar organization of the cerebral cortex and basement membrane were otherwise preserved at E18.5 (Fig. 3C, lower panel). In contrast, severe cerebral abnormalities including disruption of the pial basement membrane structure, abnormal migration of neural cells, and fused cerebral hemispheres were observed in *Emx1*-*fukutin*-cKO mice at E18.5 (Fig. 3D, lower panel). Notably, both strains exhibited defective glycosylation of α-DG at E18.5 (Fig. 3C and 3D, upper panel). We also confirmed the glycosylation state of α-DG during these embryonic stages by Western blotting (Fig. 4). In *Nestin*-*fukutin*-cKO mice, although normal glycosylation of α-DG was observed at E13.5, analysis using the IIH6 antibody against the functionally glycosylated form of α-DG revealed defects at E18.5 (Fig. 4A). We also observed decreases in molecular weight due to the loss of functional glycans using an antibody against the α-DG core protein, further suggesting abnormal glycosylation of α-DG. No apparent differences in the molecular weight of α-DG were observed between *Nestin*-*fukutin*-cKO mice and littermate controls at E13.5, indicating an almost complete absence of abnormal glycosylation. In *Emx1*-*fukutin*-cKO mice, abnormal glycosylation of α-DG was accompanied by decreases in molecular weight at E13.5 (Fig. 4B); however, experiments using IIH6 antibody detected functional glycosylation of α-DG weakly, indicating α-DG glycosylation had not been completely eliminated at this stage. In *Emx1*-*fukutin*-cKO mice, defects in α-DG glycosylation were observed at E18.5, similar to findings observed in *Nestin*-*fukutin*-cKO mice. These results suggest that the extent of abnormal α-DG glycosylation had progressed further in *Emx1*-*fukutin*-cKO mice than in *Nestin*-*fukutin*-cKO mice at E13.5, in accordance with the results of immunofluorescence analysis.

To further examine the correlation between the glycosylation state of α-DG and phenotypic severity in the developing brain, we evaluated two additional mouse models of dystroglycanopathy. *Fukutin*^Hp^ mice carry the founder retrotransposal insertion in the *fukutin* gene. Unlike FCMD, this condition is characterized by a lack of muscular involvement due to residual laminin-binding activity of α-DG (35). Mice of the *Large*^myd^ strain are characterized by spontaneous mutation of the *Large* gene and thus lack the ligand-binding moiety of α-DG from the beginning of development (36). As in *fukutin*-cKO mice, we performed immunofluorescence analysis of these two strains at E13.5 and E18.5.

**Figure 5.**
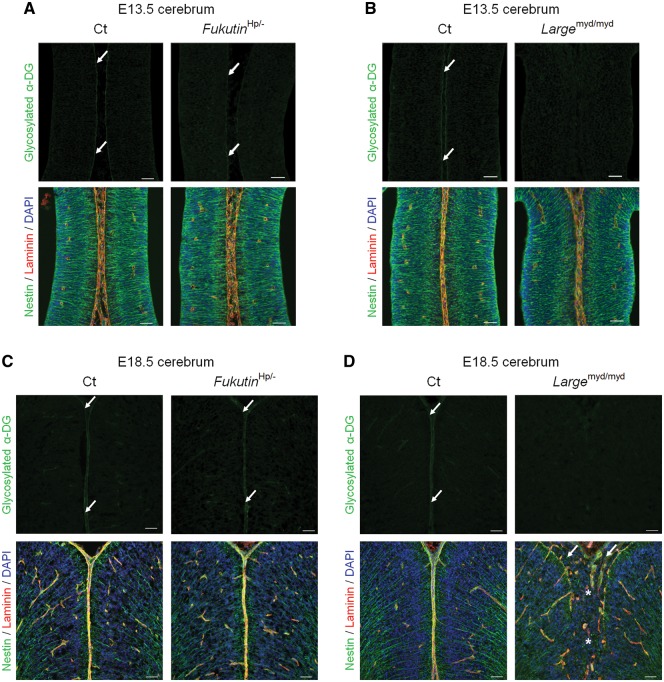
Dystroglycan glycosylation and phenotypic correlation during brain development in other mouse models of dystroglycanopathy. (**A**, **B**) Immunofluorescence analysis of the developing cortex at E13.5. (A, upper panel) *Fukutin*^Hp/-^ mice demonstrated residual α-DG glycosylation at the glia limitans (arrow). (B, upper panel) α-DG glycosylation was defective in *Large*^myd/myd^ mice. (A and B, lower panel) Both strains exhibited almost normal brain structure regardless of α-DG glycosylation state. (**C**, **D**) Immunofluorescence analysis of the developing cortex at E18.5. (C, upper panel) Glycosylation of α-DG was observed at the glia limitans in *Fukutin*^Hp/-^ mice (arrow). (D, upper panel) Glycosylation of α-DG was consistently defective in *Large*^myd/myd^ mice. (C, lower panel) Almost no structural defects were observed in *Fukutin*^Hp/-^ mice. (D, lower panel) Diffuse and severe brain malformations were observed in *Large*^myd/myd^ mice. Cerebral hemispheres were widely fused (asterisk), and the glia limitans-basement membrane complex was diffusely dissociated in *Large*^myd/myd^ mice, similar to findings observed in *Emx1*-*fukutin*-cKO mice. Many ectopic cells were observed in the subarachnoid space (arrow). Scale bars = (A, B, C, D) 50 μm.

**Figure 6.**
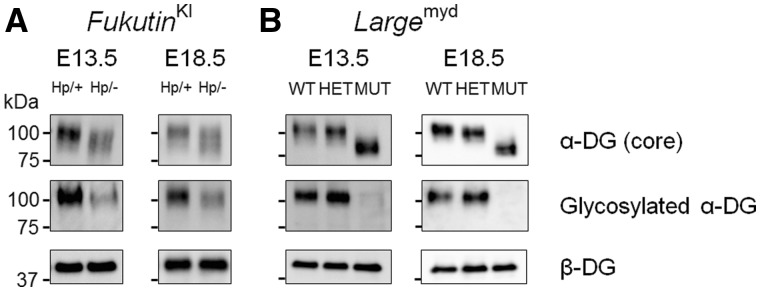
Western blotting analysis in the fetal brains of other mouse models of dystroglycanopathy. (**A**) *Fukutin*^Hp^ and (**B**) *Large*^myd^ strains were analysed at E13.5 and E18.5, respectively. We used β-DG as a loading control.

*Fukutin* ^Hp/-^ mice, which correspond to compound heterozygous mutation in human patients, demonstrated reduced yet detectable levels of normally glycosylated α-DG at E13.5 (Fig. 5A, upper panel). In contrast, normally glycosylated α-DG was defective in the developing cortex of *Large*^myd/myd^ mice at E13.5 (Fig. 5B, upper panel). In accordance with findings observed in the *fukutin*-cKO mouse strains, both strains demonstrated almost normal brain structure regardless of α-DG glycosylation state, when examined via light microscopy at this stage (Fig. 5A and B, lower panel). Interestingly, *Fukutin*^Hp/-^ mice demonstrated almost no structural defects even at E18.5 (Fig. 5C, lower panel). However, *Large*^myd/myd^ mice exhibited severe brain abnormalities similar to those observed in *Emx1*-*fukutin*-cKO mice at E18.5 (Fig. 5D, lower panel). It is noteworthy that normally glycosylated α-DG was weakly positive in *Fukutin*^Hp/-^ mice at E18.5, whereas normally glycosylated α-DG was defective in *Large*^myd/myd^ mice at this stage (Fig. 5C and D, upper panel). The glycosylation state of α-DG at these embryonic stages was confirmed by Western blotting (Fig. 6). In *Fukutin*^Hp/-^ mice, glycosylated α-DG was consistently weakly positive at both E13.5 and E18.5 (Fig. 6A), while glycosylated α-DG was consistently negative at both stages in *Large*^myd/myd^ mice (Fig. 6B), in accordance with the results of immunofluorescence analysis. Previous studies have reported that *fukutin* mRNA was already detectable at E9.5 in the neuroepithelium of the forebrain and hindbrain by *in situ* hybridization (37). We also confirmed the presence of *fukutin* and *Large* transcripts in the brains of fetal mice at E13.5 (Supplementary Material, Fig. S3). Taken together, these findings suggest that the glycosylation state of α-DG at E13.5, when cortical dysplasia has not yet become apparent, influences subsequent pathological severity in the brain.

### Severe cortical dysplasia is prevented by gene delivery during the middle stages of brain development

**Figure 7.**
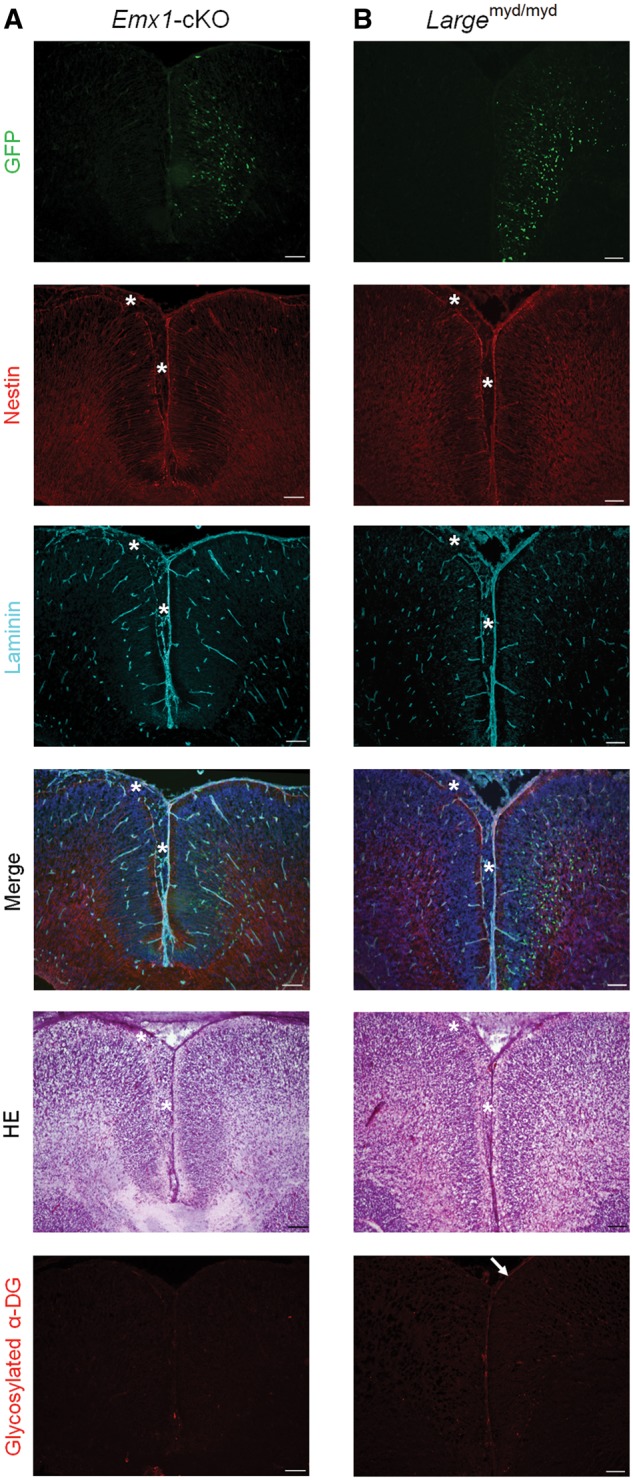
Gene rescue during the middle stage of brain development prevents severe cortical dysplasia. (**A**) Phenotypic rescue in *Emx1*-*fukutin*-cKO mice at E18.5 via delivery of the *fukutin* gene into the brains of fetal mice at E12.5. Green fluorescent protein (GFP) expression was observed in the plasmid-treated hemisphere (right hemisphere). The glia limitans-basement membrane complex was intact in the right hemisphere. In the left hemisphere, the glia limitans-basement membrane complex was diffusely dissociated, and many ectopic cells were observed in the subarachnoid space (asterisk). (**B**) Phenotypic rescue in *Large*^myd/myd^ mice at E18.5 via delivery of the *Large* gene into the brains of fetal mice at E12.5. Following gene delivery, severe cortical dysplasia was prevented in *Large*^myd/myd^ mice. Scale bars = (A, B) 100 μm.

We performed gene rescue experiments to determine whether cortical dysplasia can be prevented by the restoration of α-DG glycosylation. We used *in utero* electroporation to introduce the expression plasmid vectors into neural progenitor cells in the ventricular zone (38). Since *Emx1*-*fukutin*-cKO and *Large*^myd/myd^ mice demonstrated diffuse and severe brain malformations at E18.5 (Fig. 3D and 5D), respectively, these two strains were used for rescue experiments. Following gene delivery into the brains of fetal mice at E12.5, the extents of phenotypic recovery in these mice were analysed at E18.5. Successful gene delivery was confirmed by green fluorescent protein (GFP) expression in migrating neurons (39) in the hemisphere into which plasmids had been introduced (i.e. right hemisphere). We observed restoration of α-DG glycosylation following gene delivery: In *Large*^myd/myd^ mice, functionally glycosylated α-DG was clearly detected in the right hemisphere (Fig. 7B), while restoration of α-DG glycosylation was weak in *Emx1*-*fukutin*-cKO mice (Fig. 7A). The glia limitans-basement membrane complex was reasonably dissociated in the non-plasmid-treated hemisphere (i.e. left hemisphere). Surprisingly, severe cortical dysplasia in *Emx1*-*fukutin*-cKO (Fig. 7A) or *Large*^myd/myd^ mice (Fig. 7B) at E18.5 was prevented by the delivery of the *fukutin* or *Large* gene into the brains of fetal mice at E12.5, respectively.

## Discussion

In the present study, we analysed four distinct mouse models of dystroglycanopathy that replicate the heterogeneity of brain pathology in CMDs. Our results indicated that the glycosylation state of α-DG during brain development may influence the severity of subsequent brain pathology, suggesting that spatiotemporal persistence of functionally glycosylated α-DG is crucial for normal brain development during the fetal stage.

We first generated CNS-selective *Nestin-fukutin-*cKO mice. In accordance with the findings of previous studies (13,31,40–42), immunofluorescence analysis revealed that the mechanism underlying the development of brain abnormalities involves damage to the basement membrane and disorganization of glial cells (i.e. radial glia/Bergmann glia). In the cerebrum, radially migrating neurons migrated beyond the marginal zone due to misdirection by scaffolds composed of radial glia. In contrast, external granule cells were unable to migrate inwardly from the surface of the cerebellar cortex due to disorganization of Bergmann glia fibers.

Although abnormal glycosylation of α-DG in the brains of adult *Nestin*-*fukutin*-cKO mice was similar to that observed in FCMD (43), brain malformations in *Nestin*-*fukutin*-cKO mice were focal and much milder than those typically observed in human patients with FCMD. Since CNS-selective Cre expression driven by *Nestin* promoter is reported to begin at E10.5 (44), we suspected that residual normally glycosylated α-DG during the early stages of brain development may prevent the development of severe malformations. We speculated that normally glycosylated α-DG and originally translated fukutin protein may remain at this stage, even when *de novo* transcription/translation of fukutin protein is disrupted due to Cre-LoxP recombination at E10.5, in *Nestin*-*fukutin*-cKO mice. In addition, as fukutin is an Rbo5P transferase (18), residual fukutin protein may be sufficient for enzymatic modification of α-DG. Thus, total replacement of normally glycosylated α-DG with abnormally glycosylated α-DG may require a certain amount of time. In this context, it is noteworthy that *dystroglycan*-cKO mice created using the same *Nestin*-*Cre* transgene (*Nestin*-*DG*-cKO) demonstrate a much more severe phenotype than *Nestin*-*fukutin*-cKO mice (12). In contrast to functional deletion of dystroglycan in *Nestin*-*fukutin*-cKO mice, Cre-mediated deletion of the *dystroglycan* gene is unrelated to the glycosylation process in *Nestin*-*DG*-cKO mice, which may explain in part the phenotypic difference between these cKO mice.

We then analysed brain development in another strain of CNS-selective *fukutin*-cKO mice, *Emx1*-*fukutin*-cKO, which has been reported to drive sufficient recombination of the target gene in dorsal telencephalic neural stem cells beginning at E9.5 (34) and achieve almost complete recombination as early as E12.5 (33). Such mice exhibited severe cerebral abnormalities including disruption of the pial basement membrane structure, abnormal migration of neural cells, and fused cerebral hemispheres. Previous studies have reported phenotypic variation between *DG*-cKO mouse strains developed using different *Cre* transgenes (11,12). *DG*-cKO mice developed using *Nestin*-*Cre* transgene (*Nestin*-*DG*-cKO) exhibit earlier and more severe disruption of neuronal migration than *DG*-cKO mice developed using *GFAP*-*Cre* transgene (*GFAP*-*DG*-cKO). Cre expression driven by *GFAP* promoter is first detected in the dorsal and medial telencephalon at E13.5 (45), while Cre expression driven by *Nestin* promoter is first detected at E10.5 (44). Thus, earlier and broader expression of Cre in the brains of *Nestin*-*DG*-cKO mice may contribute to the more severe pathology observed in these mice, relative to *GFAP*-*DG*-cKO mice. Similar findings have been observed in protein O-mannosyltransferase 2 (*POMT2*)-cKO mice developed using *Emx1*-*Cre* or *GFAP*-*Cre* transgenes, respectively (41). Phenotypic heterogeneity between *fukutin*-cKO mouse strains may be due to later and insufficient *Cre* transgene expression when driven by the *Nestin* promoter rather than the *Emx1* promoter, as well as residual enzymatic activity of fukutin protein.

In addition to *fukutin*-cKO mouse strains, we further evaluated two mouse models of dystroglycanopathy. In contrast to *fukutin*-cKO strains, which exhibit normal glycosylation of α-DG until *Cre*-mediated *fukutin* deletion, these strains are characterized by either weak residual glycosylation of α-DG (*Fukutin*^Hp^ strain) or abnormal glycosylation of α-DG (*Large*^myd^ strain) from the beginning of development. These four distinct mouse models replicated the clinical heterogeneity of brain pathology observed in patients with dystroglycanopathy. Immunofluorescence analysis of the developing cortex revealed that the glycosylation state of α-DG at E13.5, when cortical dysplasia has not yet become apparent, influences subsequent pathological severity in the brain. Thus, the severity of brain abnormalities may depend on the degree of residual α-DG glycosylation during the fetal stage, possibly contributing to phenotypic heterogeneity. The findings of our gene rescue experiments further support this hypothesis.

Surprisingly, severe cortical dysplasia of *Emx1*-*fukutin*-cKO or *Large*^myd/myd^ mice at E18.5 was prevented by the delivery of the *fukutin* or *Large* gene into the brains of fetal mice at E12.5, respectively. Our results demonstrated functional restoration of α-DG and amelioration of severe brain malformations in both strains. Restoration of α-DG glycosylation was weaker in *Emx1*-*fukutin*-cKO than in *Large*^myd/myd^ mice, possibly due to functional differences between fukutin and Large as glycosyltransferases. Since extension of the ligand-binding moiety of α-DG is regulated by the enzymatic activity of Large (21), delivery of the *Large* gene may induce the hyper-extension of this moiety, which may enhance the reactivity of the antibody against glycosylated α-DG. Previous studies have reported that even partial restoration of α-DG glycosylation is sufficient to maintain muscle cell integrity in mouse models of dystroglycanopathy (35). Our results also suggested that prevention of brain malformations in these mouse models may not require the full restoration of α-DG glycosylation; however, further studies are required to determine the optimal timing, method, and degree of α-DG glycosylation for phenotypic rescue. Taken together, our findings indicate that the spatiotemporal persistence of functionally glycosylated α-DG may be crucial for normal brain development during the fetal stage. In addition, our analysis suggested that functional deletion of normally glycosylated α-DG in *Nestin*-*fukutin*-cKO mice may have occurred after the critical period during brain development.

The mechanisms underlying clinical heterogeneity of dystroglycanopathy (i.e. dystroglycanopathy type A-C) (23) remain poorly understood. Although such clinical heterogeneity in the brain may be solely due to residual levels of enzymatic activity and α-DG glycosylation, such factors cannot be investigated via biopsy in human patients. Our present data demonstrate the importance of the spatiotemporal roles of α-DG glycosylation during brain development, which may explain such phenotypic variations. In addition to the deleterious effects of gene mutation on enzymatic activity, other environmental factors, such as the local concentration of acceptor substrate for the affected enzymes, may also influence the residual activity of mutant enzymes, the glycosylation state of α-DG, and subsequent disease severity. Such environmental factors may differ among individuals and/or during prenatal development. Taken together, these findings suggest that the four distinct mouse models of dystroglycanopathy utilized in the present study represent valuable tools for the investigation of pathogenesis, synapse function, and potential therapeutic strategies in future studies.

Recent advances in respiratory care and myocardial protection have increased the lifespan of many patients with muscular dystrophy. However, mental retardation and refractory epilepsy due to brain abnormalities still limit societal participation for many patients. Previously, we suggested that splicing modulation therapy with antisense oligonucleotides represents a promising strategy for the treatment of muscular symptoms in patients with FCMD (46). However, postnatal treatment may be insufficient for central nervous system impairments, as such strategies are unable to correct neuronal migration defects that have occurred during fetal development. In contrast, dystroglycan is present at inhibitory synapses and has been reported to regulate the plasticity of mature GABAergic synapses, possibly contributing to intellectual ability (47,48). As dysfunction of inhibitory synapses also increases susceptibility to epilepsy, postnatal treatment may ameliorate these disturbances. We therefore propose that fetal therapy in conjunction with postnatal treatments such as splicing modulation therapy represents a promising therapeutic strategy for FCMD.

Advances in prenatal diagnosis provide important insights into the prenatal management and treatment of congenital diseases. Although evidence remains limited, several surgical interventions for specific anatomic anomalies, such as fetoscopic laser surgery for twin-twin transfusion syndrome, have been successfully developed (49). Recently, new therapeutic strategies beyond the simple correction of structural abnormalities via surgical interventions have been explored. Of course, unknown safety risks need to be investigated before clinical application; however, *in utero* therapy toward modulating α-DG glycosylation, such as gene delivery or ribitol/CDP-ribitol supplementation, offers the possibility of treating a wide range of dystroglycanopathy. It is noteworthy that *in utero* diagnosis of Walker-Warburg phenotype during the early stage of pregnancy (i.e. the first trimester) is now possible due to advancements in transvaginal ultrasound technology (50). Early intervention prior to the progression of brain abnormalities may improve the efficacy of the proposed treatments. In conclusion, our present findings may aid in the development of novel therapeutic strategies that can be applied during fetal development to reduce or prevent brain malformation in patients with dystroglycanopathy.

## Materials and Methods

### Generation of animal models

Floxed *fukutin* allele was generated via homologous recombination for the insertion of LoxP sites flanking the second exon of the *fukutin* gene, as previously described (51). *Nestin-Cre* mice (*Nestin-Cre*^Tg(+)^) were obtained from The Jackson Laboratory. Female homozygous floxed *fukutin* mice (*fukutin*^lox/lox^) were bred to male heterozygous floxed mice expressing *Nestin-Cre* (*Nestin-Cre*^Tg(+)^, *fukutin*^lox/+^) to generate conditional *fukutin*-knockout mice (*Nestin-Cre*^Tg(+)^, *fukutin*^lox/lox^). We used *fukutin*^lox/lox^ homozygous mice with the Cre transgene as cKOs. We used *fukutin*^lox/lox^ homozygous mice without the Cre transgene and *fukutin*^lox/+^ heterozygous mice with the Cre transgene as littermate controls (WT and HET, respectively). *Emx1-Cre* knock-in mice were generated as previously described (52), and *Emx1-fukutin*-cKO mice were generated via a breeding strategy similar to that of *Nestin-fukutin*-cKO mice. Another mouse line carrying the founder retrotransposal insertion in the *fukutin* gene (*Fukutin*^Hp/-^ or *Fukutin*^Hp/+^ mice) was generated as previously reported (35). Hp represents the transgenic allele carrying the retrotransposal insertion in *fukutin*. *Fukutin*^Hp/-^ mice correspond to compound heterozygous patients, whereas *Fukutin*^Hp/+^ mice correspond to human carriers. *Large*^myd^ mice were also obtained from The Jackson Laboratory. In each experiment, male and female mice were used equally, and more than three mice of each genotype were analysed. The genotypes of these mice were determined via PCR analysis of tail DNA. Primer sequences and PCR conditions are available on request. For timed matings, the day of vaginal plug formation was considered E0.5.

Mice were maintained in accordance with the animal care guidelines of Kobe University. All mice were housed in cages (two to four mice per cage) with wood-chip bedding in an environmentally controlled room (25°C, 12-h light-dark cycle) and provided with *ad libitum* access to food and water at the animal facility of Kobe University Graduate School of Medicine. Well-trained researchers and experimental technicians with knowledge of the methods for preventing unnecessary and excessive pain handled the animals and conducted the experiments. All animal studies were approved by the Institutional Animal Care and Use and Ethical Committee of Kobe University (P151203, P150201) in accordance with the guidelines of the Ministry of Education, Culture, Sports, Science, and Technology (MEXT) and the Japan Society for the Promotion of Science (JSPS).

### Antibodies

The following antibodies were used for Western blotting and immunofluorescence experiments: mouse monoclonal antibody 8D5 against β-DG (Leica Biosystems); mouse monoclonal antibody IIH6 against glycosylated α-DG (Millipore); goat polyclonal antibody (AP-074G-C) against the C-terminal domain of α-DG core protein (35) and rat monoclonal antibody 3D7 against α-DG core protein (53); goat polyclonal anti-fukutin antibody (106G2) against full-length fukutin protein lacking the amino (N)-terminal hydrophobic domain and rabbit polyclonal anti-fukutin antibody (RY213) against CLKIESKDPRLDGIDS (46); mouse monoclonal antibody against Nestin (Millipore); rabbit polyclonal antibody against laminin (Sigma); mouse monoclonal antibody against glial fibrillary acidic protein (GFAP) (Millipore).

### Detection of endogenous fukutin protein

Detection of endogenous fukutin protein was performed using goat polyclonal anti-fukutin antibody 106G2 and rabbit polyclonal anti-fukutin antibody RY213 as previously described (46). Briefly, endogenous fukutin was enriched via immunoprecipitation using 106G2 from brain lysates in TBS (pH 7.4) containing 1% Triton X-100 and protease inhibitor cocktail (Nacalai Tesque). Fukutin expression was then detected via Western blotting using RY213.

### Dystroglycan preparation and Western blotting

Tissues were solubilized in TBS (pH 7.4) containing 1% Triton X-100 and protease inhibitor cocktail (Nacalai Tesque). Samples were centrifuged at 20, 800*g* for 10 min at 4°C, following which the solubilized fraction was collected and incubated with wheat germ agglutinin (WGA)-agarose beads (Vector Laboratories) at 4°C overnight. The beads were washed three times in TBS (pH 7.4) containing 0.1% Triton X-100. The bound proteins were eluted with TBS (pH 7.4) containing 0.1% Triton X-100 and 300 mM N-acetyl-glucosamine. Proteins were separated via SDS-PAGE on 4–15% gels, transferred to polyvinylidene difluoride (PVDF) membranes, and probed with antibodies to dystroglycan.

### Laminin overlay assay

The laminin overlay assay was performed as previously described (35). Briefly, PVDF membranes were blocked in laminin-binding buffer (LBB: 10 mM triethanolamine, 140 mM NaCl, 1 mM MgCl_2_, 1 mM CaCl_2_, pH 7.6) containing 5% non-fat dry milk, followed by incubation with 7.5 nM mouse Engelbreth–Holm–Swarm (EHS) laminin (Sigma) at 4°C overnight in LBB containing 3% BSA. Subsequently, membranes were washed and incubated with rabbit anti-laminin antibody (Sigma) at 4°C for 3 h, followed by anti-rabbit IgG–HRP at room temperature for 1 h.

### Histology and immunofluorescence analysis

For routine histology, adult mice 10–20 weeks of age were deeply anesthetized, transcardially perfused with PBS, and fixed with 4% paraformaldehyde (PFA) in PBS. Subsequently, brains were postfixed in 4% PFA-PBS solution at 4°C overnight, dehydrated in an ascending series of ethanol solutions, cleared in xylene, and then embedded in paraffin. Paraffin blocks were serially sectioned at a thickness of 8 μm using a microtome (Leica RM2135). Sections were stained with hematoxylin and eosin in accordance with standard protocols.

For immunofluorescence analysis, brains were fixed overnight with 4% PFA-PBS as described above, cryoprotected overnight with 30% sucrose in PBS, embedded in OCT compound (Tissue-Tek), and cryosectioned at a thickness of 12 μm using a cryostat (Leica CM1850). After antigen retrieval with HistoVT One (Nacalai Tesque) in accordance with the manufacturer's protocol, slides were incubated in blocking solution containing 5% goat serum in MOM Mouse Ig Blocking Reagent (Vector Laboratories) at room temperature for 1 h, following which they were incubated with primary antibodies diluted in MOM Diluent (Vector Laboratories) at 4°C overnight. Subsequently, slides were washed with PBS and incubated with Alexa Fluor 488-, 555-, or 647-conjugated secondary antibodies (Molecular Probes) at room temperature for 30 min. For nuclear staining, 4′6-diamidino-2-phenylindole (DAPI, Sigma) was added to the secondary antibody solution. Images were acquired using a BZ-9000 microscope (Keyence).

### *In utero* electroporation

*In utero* electroporation was performed as previously described (39). Plasmid DNA was microinjected into the lateral ventricle of E12.5 mouse embryos. Electroporation was performed using a square wave electroporator NEPA21 (Nepagene). Four electrical pulses (40 V, 30 ms duration at intervals of 970 ms) were delivered. Six days after electroporation (E18.5), fetal brains were fixed, cryosectioned, and immunostained as described above. The cDNAs encoding EGFP, mouse *fukutin*, or mouse *Large* were cloned into pCAG vector (pCAG-EGFP, pCAG-fukutin, or pCAG-Large, respectively) (54). Final injected dosages of plasmid DNA in approximately 2 μl TE were as follows: 1.0 μg for pCAG-EGFP vector, 5.0 μg for pCAG-fukutin vector or pCAG-Large vector. The pCAG-fukutin and pCAG-EGFP vectors were electroporated into *Emx1-fukutin*-cKO mouse embryos simultaneously, and pCAG-Large and pCAG-EGFP vectors were electroporated into *Large*^myd/myd^ mouse embryos simultaneously.

## Supplementary Material

Supplementary Material is available at *HMG* online.

## Supplementary Material

Supplementary Material
