# Supplementary material for: Temporal requirement of dystroglycan glycosylation during brain development and rescue of severe cortical dysplasia via gene delivery in the fetal stage
Source: Hum Mol Genet. 2018 Jan 19;27(7):1174–85. doi: 10.1093/hmg/ddy032 (PMC6159531; doi:10.1093/hmg/ddy032)
Supplement: Supplementary Material [file ddy032_supplementary_material.docx]

**SUPPLEMENTARY MATERIAL**

**Title:**

Temporal requirement of dystroglycan glycosylation during brain development and rescue of severe cortical dysplasia via gene delivery in the fetal stage

**Author names:**

Atsushi Sudo, Motoi Kanagawa, Mai Kondo, Chiyomi Ito, Kazuhiro Kobayashi, Mitsuharu　Endo, Yasuhiro Minami, Atsu Aiba, Tatsushi Toda

**
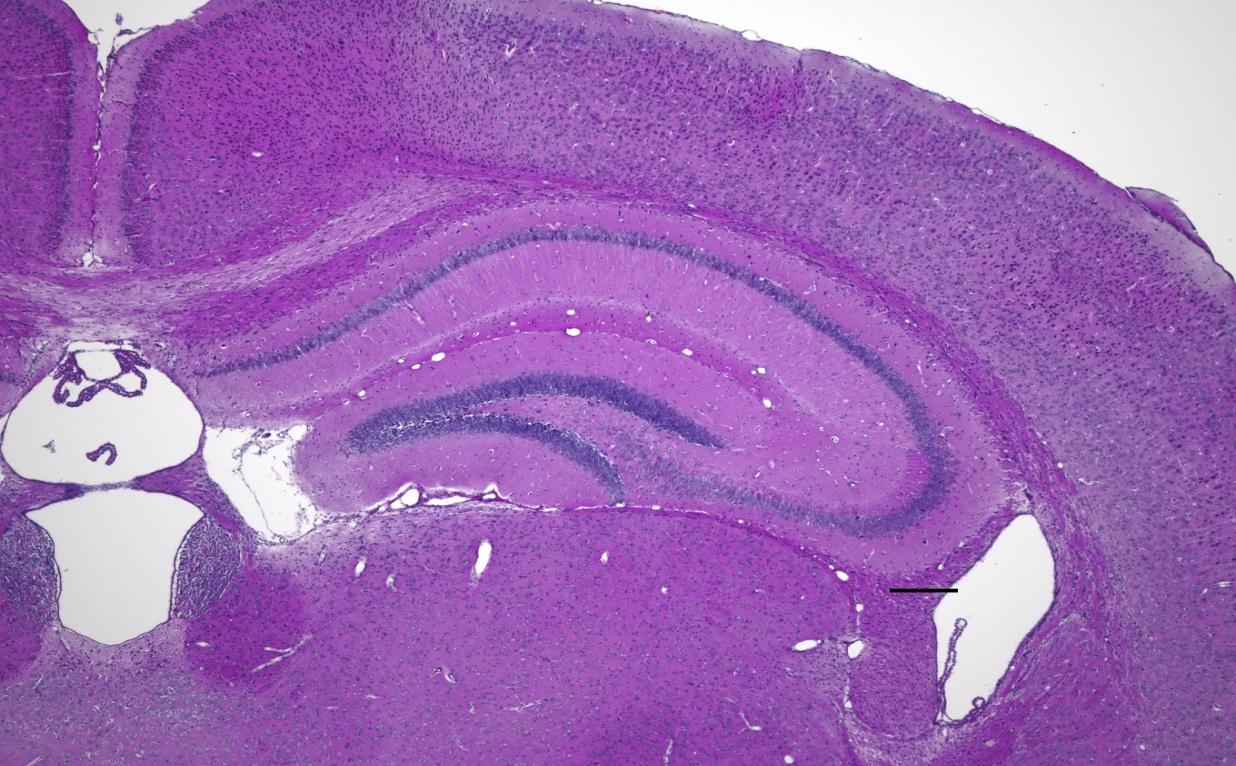
**

Ct

**
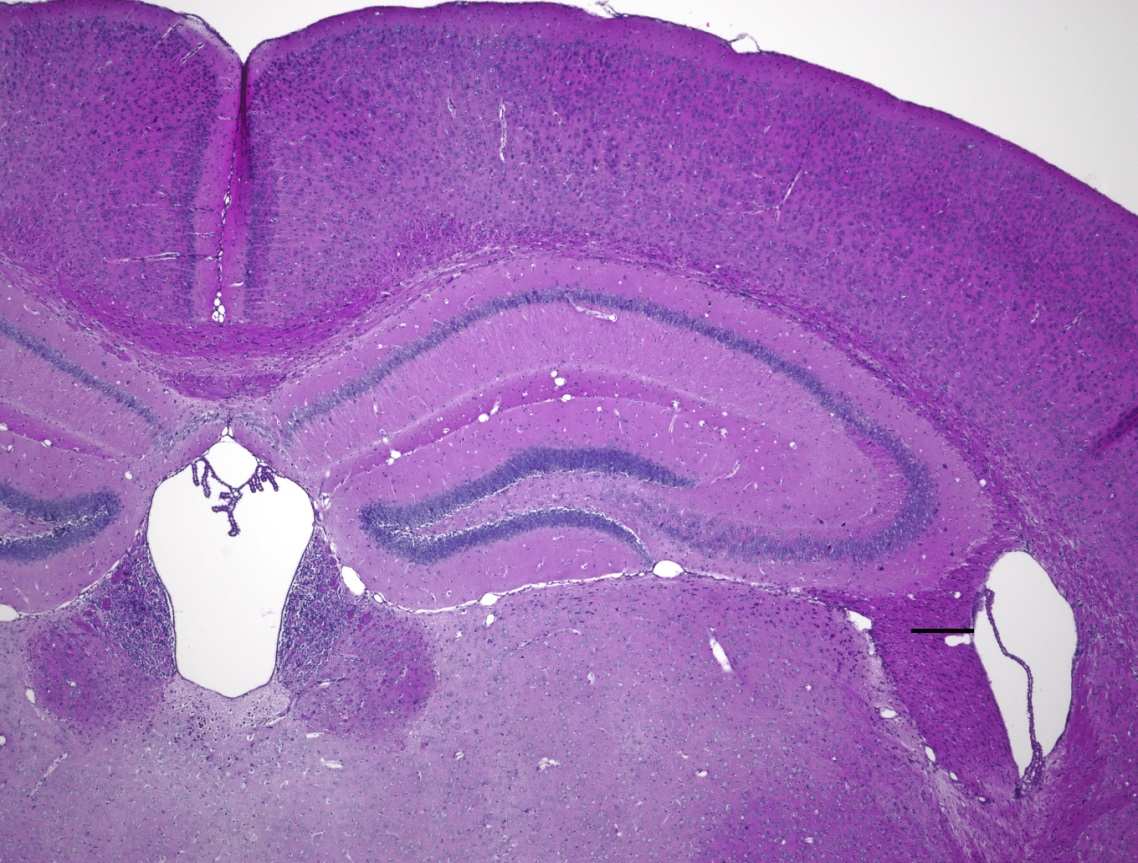
**

*Nestin*-cKO

**Figure S1. H&E staining of the hippocampus in adult *Nestin*-*fukutin*-cKO mice.**

No obvious difference was detected between cKO mice and littermate controls. Scale bars = 200 μm.

**Figure S2. Ectopic cellular infiltration through breaches of the basement membrane in *Nestin*-*fukutin*-cKO mice.**

In cKO mice, radial glia fibers were disorganized and extended into subarachnoid spaces through breaches in the basement membrane (asterisk) at E18.5. Protrusion of neurons was observed at the subarachnoid space (arrow). In contrast, glia limitans-basement membrane complex was intact and they had merged properly in littermate controls. Scale bars = 500 μm.

♨


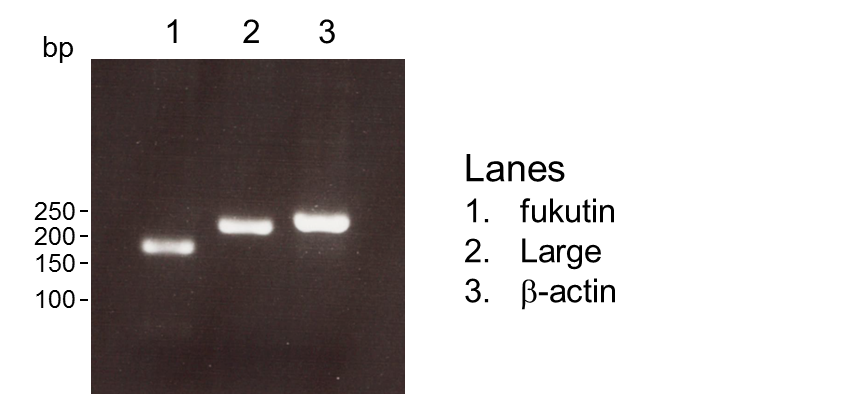


**Figure S3. RT-PCR analysis of *fukutin* and *Large* in the fetal brain of *fukutin*^lox/lox^ control mice at E13.5.**

Total RNA was isolated from the fetal brain of control mice at E13.5 using RNeasy Plus mini kit (Qiagen) and converted to cDNA using Superscript IV reverse transcriptase (Invitrogen) in accordance with the manufacturers’ protocols. Primer sequences and PCR conditions are available on request.

*Emx1*-Het


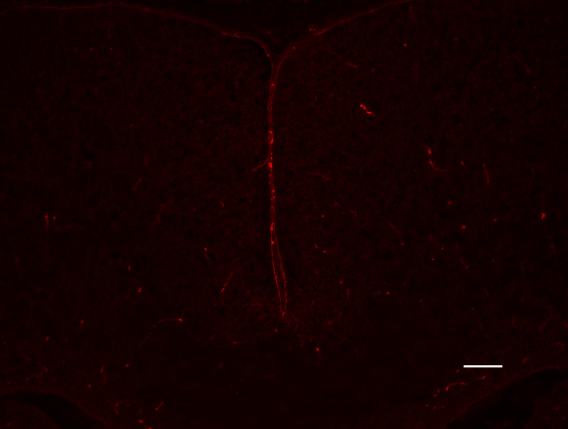


*Large*^myd/+^


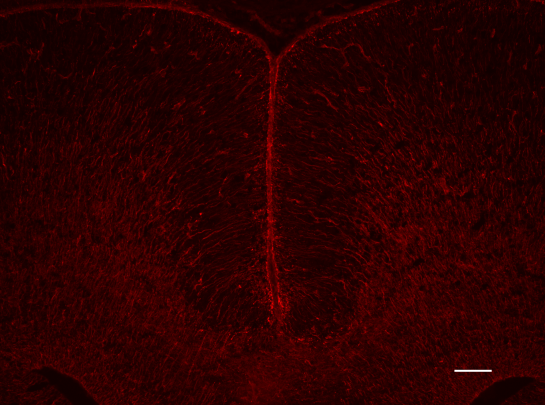


Nestin


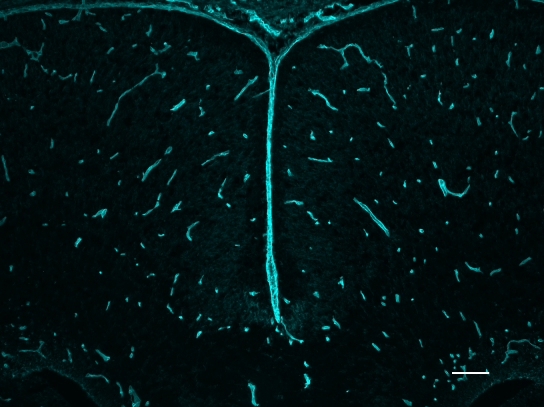


Laminin


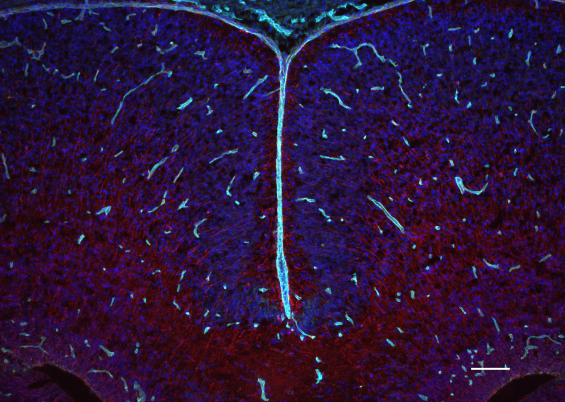


Merge

Glycosylated α-DG


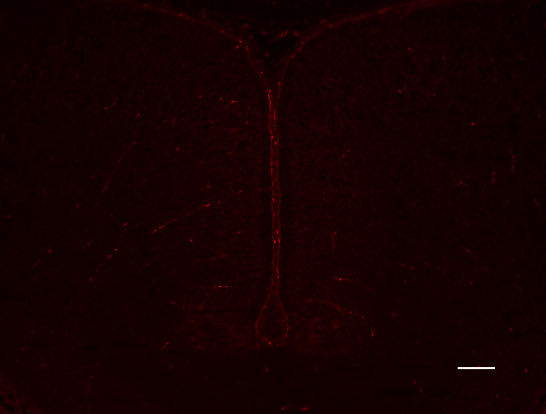

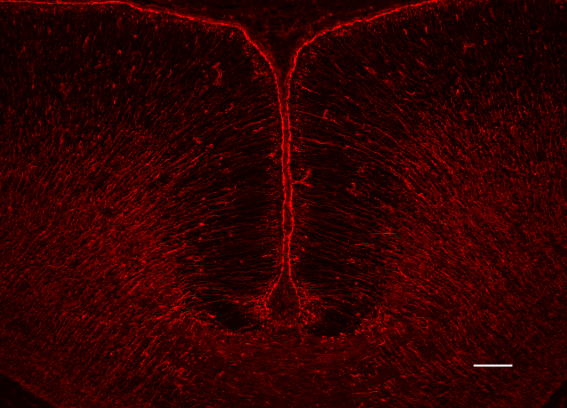

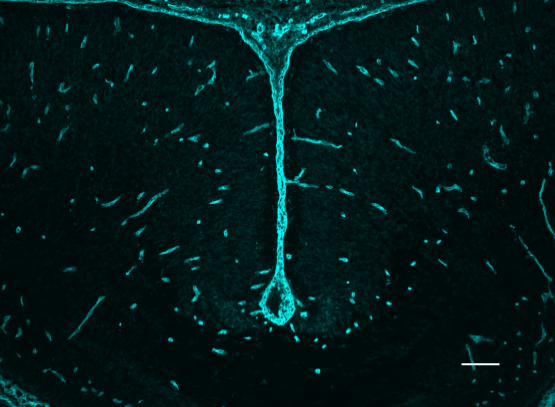

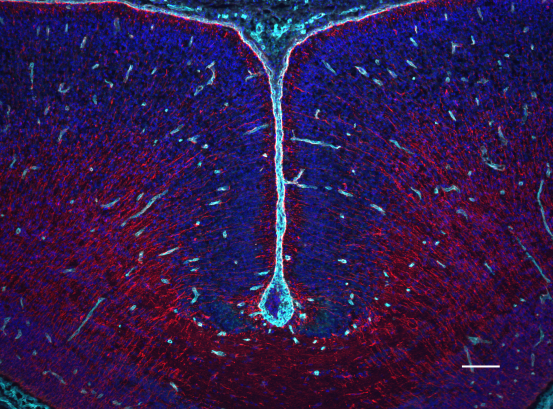


**Figure S4. Immunofluorescence analysis of the developing cortex in littermate controls at E18.5.**

Functionally glycosylated α-DG was detected at the glia limitans in these littermate controls. These images were obtained under the same experimental conditions described in the manuscript. Scale bars = 1000 μm.
